# *In situ* ruminal degradation characteristics of dry matter and crude protein from dried corn, high-protein corn, and wheat distillers grains

**DOI:** 10.1186/s40781-016-0115-3

**Published:** 2016-09-01

**Authors:** Y. H. Lee, F. Ahmadi, D. Y. Choi, W. S. Kwak

**Affiliations:** Division of Food Biosciences, College of Medical Life Sciences, Konkuk University, Chung-Ju, Chung-Buk 380-701 Korea

**Keywords:** Crude protein, Dried distillers grains with solubles, Undegraded intake protein, *In situ* rumen degradation

## Abstract

**Background:**

The continuing growth of the ethanol industry has generated large amounts of various distillers grains co-products. These are characterized by a wide variation in chemical composition and ruminal degradability. Therefore, their precise formulation in the ruminant diet requires the systematic evaluation of their degradation profiles in the rumen.

**Methods:**

Three distillers grains plus soluble co-products (DDGS) namely, corn DDGS, high-protein corn DDGS (HP-DDGS), and wheat DDGS, were subjected to an *in situ* trial to determine the degradation kinetics of the dry matter (DM) and crude protein (CP). Soybean meal (SBM), a feed with highly degradable protein in the rumen, was included as the fourth feed. The four feeds were incubated in duplicate at each time point in the rumen of three ruminally cannulated Hanwoo cattle for 1, 2, 4, 6, 8, 12, 24, and 48 h.

**Results:**

Wheat DDGS had the highest filterable and soluble A fraction of its DM (37.2 %), but the lowest degradable B (49.5 %; *P* < 0.001) and an undegradable C fraction (13.3 %; *P* < 0.001). The filterable and soluble A fraction of CP was greatest with wheat DDGS, intermediate with corn DDGS, and lowest with HP-DDGS and SBM; however, the undegradable C fraction of CP was the greatest with HP-DDGS (41.2 %), intermediate with corn DDGS (2.7 %), and lowest with wheat DDGS and SMB (average 4.3 %). The degradation rate of degradable B fraction (% h^−1^) was ranked from highest to lowest as follows for 1) DM: SBM (13.3), wheat DDGS (9.1), and corn DDGS and HP-DDGS (average 5.2); 2) CP: SBM (17.6), wheat DDGS (11.6), and corn DDGS and HP-DDGS (average 4.4). The *in situ* effective degradability of CP, assuming a passage rate of 0.06 h^−1^, was the highest (*P* < 0.001) for SBM (73.9 %) and wheat DDGS (71.2 %), intermediate for corn DDGS (42.5 %), and the lowest for HP-DDGS (28.6 %), which suggests that corn DDGS and HP-DDGS are a good source of undegraded intake protein for ruminants.

**Conclusions:**

This study provided a comparative estimate of ruminal DM and CP degradation characteristics for three DDGS co-products and SBM, which might be useful for their inclusion in the diet according to the ruminally undegraded to degraded intake protein ratio.

## Background

Dried distillers grains with solubles (DDGS), generated after the fermentation and distillation in a grain-based ethanol production [1], are an excellent and economical source of protein and energy for livestock, especially dairy and beef cattle. Although similar to the original grain, DDGS is usually richer in protein, fat, and fiber concentrations [2, 3]. Relative to SBM, DDGS co-products are generally recognized to have a proportionately higher ruminally undegraded intake protein (UIP) content. This is because most of the readily degradable protein in the grain is degraded during the fermentation process [3]. For example, the National Research Council [4] estimates that corn DDGS contains approximately 30 % crude protein, where UIP constitutes about 73 % of crude protein (CP).

The rapidly expanding ethanol industry will contribute significantly to an increased supply of DDGS at a competitive cost [5, 6]. However, considerable variation in the processing technologies among the dry-milling industries and the raw grains entering the plants, substantially alter the chemical composition and ruminal degradation properties of DDGS co-products. This necessitates a more accurate estimate of ruminal degradation kinetics of dry matter (DM) and CP fractions. Reliably determining the dynamic models of carbohydrate and protein digestion is key to gaining the maximum economic benefit from these co-products [7–9]. Therefore, to precisely formulate diets to meet, but not exceed, the protein requirement of the ruminant, there is an urgent need to more specifically characterize the protein (especially the UIP fraction) degradation kinetics of these co-products in the rumen [6, 10]. This will subsequently mitigate excessive N loss into the environment, and have economic benefits while supporting optimal animal performance [11]. Therefore, this study aims to compare *in situ* ruminal fractions, ruminal disappearance rate, and effective degradability (ED) of DM and CP in corn, high-protein corn, and wheat DDGS with those of SBM.

## Methods

### Sample preparation and chemical composition

In an attempt to collect representative samples, three different batches of each sample, on three different days throughout the months of July and August 2014, were obtained through Egreen Co. (Egreen Co. Ltd., Icheon, South Korea). Samples of HP-DDGS and corn DDGS were originated from ethanol plants in Valero’s ethanol plant in Jefferson, Wisconsin (USA) and wheat DDGS was originated from bioethanol plants located in western Canada. However, the detailed information of the DDGS co-products, including the processing conditions at the ethanol production plant, were not available for this experiment owing to the difficulty in tracing detailed international information in a practical manner.

Subsamples of feeds were mixed thoroughly and hammer-milled (Cemotec, Tecator, Sweden) to pass through a 1-mm sieve, prior to being analyzed [12] for contents of DM, CP, ether extract (EE), and crude ash. Neutral detergent fiber (NDF; with heat-stable amylase and without sodium sulfite) and acid detergent fiber (ADF) content were determined using the procedure of Van Soest et al. [13]. True protein was quantified with the precipitation of the N fractions in a 5 % trichloroacetic acid solution. Difference between CP (N × 6.25) and true protein was defined as non-protein N (NPN) [14].

### *In situ* ruminal incubations

All procedures involving animals were reviewed and approved by the Konkuk University Institutional Animal Care and Use Committee. Details for the measurements of *in situ* degradability were described earlier [15]. Briefly, each sample was hammer-milled and passed through a 2-mm sieve, pooled to form one homogeneous preparation, and then randomly divided into 12 sub-samples for the *in situ* incubations (6 for each run). Each 5-g feed sample (DM basis; particles in a size range of 63 μm to 2 mm) was placed in a Dacron bag (10 × 20 cm, 53 ± 10 μm pore size; R1020, Ankom Technology, Macedon, NY, USA), and then suspended in the rumen of three cannulated Hanwoo cattle (body weight = 440.3 ± 29.17 kg, mean ± SD), which were fed a total mixed ration containing sudangrass silage (7.4 % CP, 60 % NDF and 43 % ADF) and a concentrate mix of 14.3 % CP and 6.3 % CF. The proportion of the sample weight (DM basis) to the Dacron bag surface area was 40 mg/cm^2^. For simultaneous removal of all bags from the rumen, Dacron bags were incubated at 1, 2, 4, 6, 8, 12, 24, and 48 h and placed in the rumen in reverse order of incubation time. After the incubation was completed, bags were washed under running tap water until clear water emerged from the bag, and then dried at 55 °C for 48 h. Zero hour bags were not placed in the rumen but were subjected to the same rinsing procedures as described for rumen-incubated samples.

*In situ* degradation curves of DM and CP were fitted to the exponential model described by Ørskov and McDonald [16]. The ruminal ED of DM and CP, assuming two fractional passage rates (*K*_p_B) from the rumen of 0.06 and 0.025 h^−1^, was calculated according to the following equation [16]:

1 $$ \mathrm{E}\mathrm{D}=\mathrm{A}+\mathrm{B}\left[{K}_{\mathrm{d}}\mathrm{B}/\left({K}_{\mathrm{d}}\mathrm{B}+{K}_{\mathrm{p}}\mathrm{B}\right)\right], $$

where A = 53-μm filterable and soluble fraction, which was washed out without rumen incubation, B = potentially degradable fraction, which was degraded exponentially, *K*_d_B = the degradation rate of degradable B fraction, and *K*_p_B = the passage rate of degradable B fraction. The ED of CP was considered to be DIP, and estimated UIP was calculated as [100 − % DIP] [17]. The undegradable fraction (C) was estimated as [1 – (A + B)].

### Statistical analysis

*In situ* data for each feed were a mean of 12 observations, which were obtained over the course of two consecutive runs at two different days. The experimental design was 2 consecutive incubations × 4 feeds × 3 animal replicates × 2 sample replicates, giving a total of 48 observations. The data were analyzed using the PROC MIXED of SAS (version 9.1; SAS Inst., Inc., Cary, NC, USA), where feed sources were considered fixed effects and incubation run in the rumen was assumed to be random effect. The animal data were averaged prior to statistical analysis. The model used for the analysis was: Y_*ij*_ = *μ* + F_*i*_ + R_*j*_ + e_*ij*_, where, Y_*ij*_ = the observation of the dependent variable *ij*; *μ* = the overall mean of Y; F_*i*_ = the effect of feed (*i* = 4), R = the effect of incubation run as replications (*j* = 2), and e*ij* = the random error associated with the observation *ij*. Mean separation was performed using the Tukey’s multiple range test at 5 % significance level.

## Results

### Chemical composition of the tested feeds

The chemical composition of the DDGS co-products and SBM is presented in Table 1. As proteinaceous feed, the tested DDGS contained less CP (33–39 %), more EE (1.7–3.9 %), more fiber (52–58 % NDF and 30–35 % ADF), and less crude ash (1.4–6.5 %) compared to the values in SBM.

**Table 1:** Chemical composition of soybean meal and dried distillers grains co-products^a,b^

Item (DM basis)	Soybean meal	Wheat DDGS	Corn DDGS	HP-DDGS	SE
Dry matter, %	92.4	86.5	86.5	90.0	0.05
Organic matter	92.8	93.5	95.5	98.6	0.26
Crude protein (CP)	52.2	38.1	33.3	38.8	0.38
True protein, % of CP	89.9	84.6	91.9	93.0	1.33
Non-protein nitrogen, % of CP	10.1	15.4	8.10	7.00	1.33
Ether extract	0.77	2.63	3.92	1.66	0.20
Neutral detergent fiber (NDF)	34.5	56.6	52.3	58.4	2.32
Acid detergent fiber	12.9	29.7	31.9	35.3	0.25
Crude ash	7.22	6.54	4.52	1.41	0.26

^a^ *DDGS* distillers dried grains with solubles, *HP-DDGS* high-protein corn DDGS

^b^Means of 3 observations

### Ruminal degradation kinetics of DM and CP

The mean DM degradation variables across the feeds are presented in Table 2. The mean for the filterable and soluble DM A fraction ranged from 13.6 to 37.2 % and was the highest with wheat DDGS, intermediate with corn DDGS and SBM, and the lowest with HP-DDGS (*P* < 0.001), whereas the mean of the degradable B fraction of DM ranged from 49.5 to 69.0 % and was the lowest with corn and wheat DDGS, intermediate with HP-DDGS and the highest with SBM (*P* < 0.001). The mean values of the C fraction, or ruminally undegradable DM, was highest for HP-DDGS, followed by corn DDGS, and then wheat DDGS and SBM (*P* < 0.001).

**Table 2:** Dry matter (DM) and crude protein (CP) degradation characteristics of soybean meal and dried distillers grains co-products^1, 2^

Item	Soybean meal	Wheat DDGS	Corn DDGS	HP-DDGS	SE	*P* value
DM (%)	92.4	86.5	86.5	90.0	0.1	–
DM fractions (% of DM)						
53-μm filterable and soluble A fraction	28.4^b^	37.2^a^	28.9^b^	13.6^c^	0.6	<0.001
Degradable B fraction	69.0^a^	49.5^c^	51.0^c^	55.3^b^	1.4	<0.001
Undegradable C fraction	2.6^d^	13.3^c^	20.1^b^	30.9^a^	1.2	<0.001
*K*_d_B^3^ (% h^−1^)	13.3^a^	9.1^b^	5.3^c^	5.0^c^	1.0	<0.001
CP (%)	52.2	38.1	33.3	38.8	0.4	–
CP fractions (% of CP)						
53-μm filterable and soluble A fraction	8.9^c^	26.5^a^	19.7^b^	9.0^c^	1.9	<0.001
Degradable B fraction	87.2^a^	67.8^b^	50.6^bc^	49.8^c^	4.0	0.001
Undegradable C fraction	3.9^c^	4.7^c^	29.7^b^	41.2^a^	3.3	<0.001
*K*_d_B^4^ (% h^−1^)	17.6^a^	11.6^b^	4.9^c^	3.9^c^	0.6	<0.001

^1^ *DDGS* distillers dried grains with solubles, *HP-DDGS* high-protein corn DDGS

^2^Means of 12 observations

^3^ *K*_d_B = degradation rate of degradable B fraction

^a−d^Means with different superscripts within the same row differ (*P* < 0.05)

Mean values of ruminal CP degradation variables are presented in Table 2. The mean for filterable and soluble A fraction of CP was greatest for wheat DDGS (26.5 %), intermediate on corn DDGS (19.7 %), and lowest for HP-DDGS and SBM (average 9.0 %) (*P* < 0.001). The highest and lowest degradable B fraction of CP was recorded for SBM (87.2 %) and HP-DDGS (49.8 %), respectively. The range of the C fraction for CP was from 4.7 % for wheat DDGS to 41.2 % for HP-DDGS.

Mean percentages for the degradation rate of the degradable B fraction for DM and CP, within 48 h of incubation, differed considerably among the 4 feeds, ranging from 5.0 to 13.3 % h^−1^ (SE = 0.97; *P* < 0.001) and 3.9 to 17.6 % h^−1^ (SE = 0.64; *P* < 0.001), respectively, with the lowest rate for corn and HP-DDGS being recorded and the highest rate for SBM (Table 2).

The ED of DM was higher for wheat DDGS and SBM than for corn DDG and HP-DDGS (Table 3). The ED of CP, assuming a passage rate of 0.06 h^−1^, showed that HP-DDGS was the lowest DIP source, followed by corn DDGS (42.5 %), and then SBM and wheat DDGS (72.6 %) (Table 3).

**Table 3:** *In situ* effective degradability of dry matter (DM) and crude protein (CP) at two passage rates^1, 2^

Item	Soybean meal	Wheat DDGS	Corn DDGS	HP-DDGS	SE	*P* value
Effective degradability of DM						
*K*_p_B = 0.025, h^−1^	86.5^a^	76.0^b^	63.5^c^	50.5^d^	0.5	<0.001
*K*_p_B = 0.06, h^−1^	75.9^a^	67.0^b^	52.8^c^	38.7^d^	0.9	<0.001
Effective degradability of CP						
*K*_p_B = 0.025, h^−1^	85.3^a^	82.3^a^	53.2^b^	39.3^c^	1.4	<0.001
*K*_p_B = 0.06, h^−1^	73.9^a^	71.2^a^	42.5^b^	28.6^c^	1.5	<0.001

^1^ *DDGS* distillers dried grains with solubles, *HP-DDGS* high-protein corn DDGS

^2^Means of 12 observations

Effective degradability = A + B [*K*_d_B/(*K*_d_B + *K*_p_B)], where A = 53-μm filterable and soluble fraction, B = degradable fraction, C = undegradable fraction, *K*_d_B = degradation rate of degradable B fraction, and *K*_p_B = ruminal passage rate of degradable B fraction

^a−d^Means with different superscripts within the same row differ (*P* < 0.05)

**Fig. 1:**
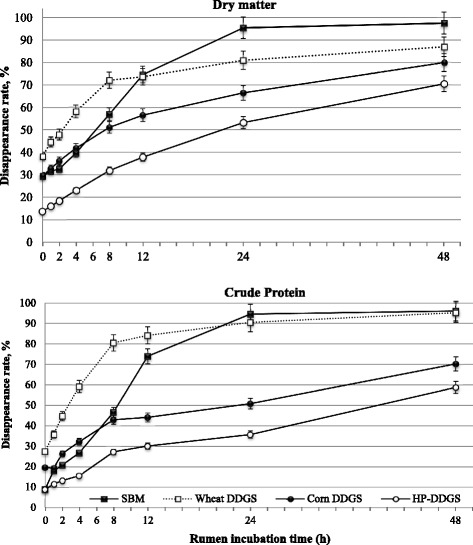
In situ dry matter and crude protein disappearance rate of the tested feeds according to rumen incubation time. Error bars indicate SE

As a function of residence time in the rumen, the amount of DM and CP disappearance for the test feeds is presented in Fig. 1. Among the feeds, wheat DDGS and SBM proteins were rapidly disappeared; approximately 84 and 74 % of their CP fraction was disappeared during the first 12 h of incubation, respectively, thereby showing a steep increase in disappearance during the first hours of incubation. Both DM and CP disappearance after 48 h of incubation followed the same trend, with lower (*P* < 0.001) and higher rates of disappearance resulting from HP-DDGS and SBM, or wheat DDGS, respectively.

## Discussion

### Chemical composition

The large difference in CP content across the 4 feeds was comparable to those mentioned in published reports [18–20]. The greater CP content found in wheat DDGS as compared to corn DDGS in the present study was in agreement with two previous reports [8, 21], which attributed the difference to a higher CP content in wheat. In general, the results of this experiment in chemical composition of the feeds were comparable to the mean reported values of the three studies with corn and wheat DDGS [8, 22, 23]. These studies reported that wheat-based DDGS is characterized by a higher concentration of CP, 42.7 *vs*. 31.4 %, and similar NDF, 34.7 and 37.5 %, respectively.

Among the different types of DDGS co-products, HP-DDGS is generally recognized for its reduced concentration of EE, ADF, and NDF, because during its production process much of the fiber is removed in de-hulling [24]. However, in this experiment HP-DDGS had a proportionately higher concentration of dietary fiber and EE as compared to corn or wheat DDGS (Table 1). The difference in CP, EE, and fiber concentrations among the DDGS co-products is largely influenced by their concentrations in the original grain and the efficiency of starch-to-ethanol fermentation [17, 21].

### *In situ* degradation kinetics

Consistent with the results of this experiment, Li et al. [21] compared the CP degradation kinetics of wheat and corn DDGS, and reported that the A and B fractions were approximately 3.8 and 23.3 % higher for wheat than for corn DDGS, but the *K*_d_ of the B fraction was much lower for wheat DDGS (2.7 % h^−1^) than for corn DDGS (7.2 % h^−1^). The range of fraction A in the DDGS co-products [corn, wheat, and HP-DDGS, from 9.0 to 26.5 %, respectively] is in contrast to the values reported by Kleinschmit et al. [25] for various sources of DDGS (15.9 to 19.7 %), and the values reported by Mjoun et al. [17] for corn DDGS, reduced-fat corn DDGS, and HP-DDGS (ranging from 11.1 to 18.4 %).

The combination of fractions A and B was 96.1, 70.3, and 94.3 % for SBM, HP-DDGS, and wheat DDGS, respectively, which is consistent with values reported for the same feeds in a recent experiment by Maxin et al. [18], but not for HP-DDGS, which was estimated to be 87.0 %. Mjoun et al. [17], studying the *in situ* CP degradability of SBM and DDGS, reported that the B fraction for CP was greatest with SBM and HP-DDGS (average 88.0 %), followed by corn DDGS (76.8 %). These values are comparable to the results of this study for SBM; however, they are considerably higher for the DDGS co-products. The degradation rate of the B fraction for CP was reported as 11.8 % h^−1^ for SBM, which is lower than that recorded in this experiment (17.6 % h^−1^).

Cao et al. [26] found that there is a linear relationship in the soluble A fraction between CP and the amount of solubles added to DDG during the production process, which may explain the variation in the A fraction among the DDGS co-products found in this experiment with those reported in the literature. The data for the undegradable C fraction on CP is in contrast with the study by Kleinschmit et al. [25], which reported that the C fraction varied from 2.1 % for high-quality DDGS to 18.7 % for a heat-damaged DDGS.

Consistent with this study’s results, the two previous studies by Nuez-Ortín and Yu [8] and Chrenková et al. [27] reported that wheat DDGS had higher *in situ* CP degradability than corn DDGS. The greater protein degradability of SBM in the rumen, compared to the DDGS products, has already been extensively studied. Aines et al. [28] summarized several experiments and reported that the DDGS protein was 1.8-fold less degradable compared with SBM protein. In an *in situ* trial by Salem et al. [29], 95.6 and 95.3 % of SBM DM and CP degraded at a rate of 14.7 and 16.7 % h^−1^, respectively, similar to the values we recorded in this study.

### Effective degradability

The higher ED of DM for wheat DDGS and SBM than for corn DDG and HP-DDGS might be partially explained by its high soluble fraction [28.4 % and 37.2 % of DM for wheat DDGS and SBM *vs*. 28.9 and 13.6 % of DM for corn DDGS and HP-DDGS, respectively], and the higher degradable B fraction of DDGS co-products [49.5 % and 69.0 % of DM for wheat DDGS and SBM vs. 51.0 % and 55.3 % of DM for corn DDGS and HP-DDGS, respectively].

The estimated UIP, assuming the passage rate of 0.06 h^−1^, ranged from 26.1 % for SMB to 71.4 % for HP-DDGS, which indicates that HP-DDGS is a good source of UIP for ruminants. This compares with previous studies reporting that UIP constituted approximately 55.2 % of CP in HP-DDGS [30] and 55 % of CP in corn DDGS [31]. The average amount of UIP (% of CP) for corn DDGS, assuming *K*_p_B = 0.06, h^−1^, was estimated to be 57.5 %, which is comparable to the value (54.9 %) reported in the National Research Council’s (NRC) Nutrient Requirements of Beef Cattle [32].

The amount of UIP in this study (assumed *K*_p_B = 0.06 h^−1^) differed from the results reported by Mjoun et al. [17] (*K*_p_B = 0.06 h^−1^): 26.1 *vs*. 32.3 % of CP for SBM, 57.5 *vs*. 52.3 % of CP for corn DDGS, and 71.4 *vs*. 54.5 % of CP for HP-DDGS, in the present study vs. Mjoun et al. [17], respectively. The amount of UIP reported by Li et al. [21] assuming *K*_p_B = 0.06 h^−1^, is considerably higher for wheat DDGS as compared to this experiment (a 22-percentage-unit difference), however the value reported for corn DDGS was comparable to the current observation.

Consistent with previous reports, this study reaffirmed that DDGS co-products have a high concentration of ruminally undegraded intake protein (by-pass protein), which could be due to much of their protein being heat-denatured yeast that was heated during the distillation and concentration process [33]. This results in the proteins being resistant to lyses and ruminal microbial degradation [3].

Endosperm is mainly composed of zein protein, which is known to be resistant to ruminal degradation [34, 35]. During the production process of HP-DDGS, a large proportion of proteins originate from endosperm, which might explain why HP-DDGS had the highest proportion of UIP compared to other DDGS co-products.

To meet the requirements for metabolizable protein, and minimize N excretion, the dietary protein must be divided into DIP and UIP fractions, which requires a precise estimation of protein degradation in the rumen for the feed ingredients included in the diet [10]. Therefore, the estimates of UIP reported in this experiment might be particularly useful for farmers and feed manufactures to more precisely formulate the diet.

The variations observed in the results of this study compared with those reported in the literature might be explained in part by differences in feed particle size or laboratory-to-laboratory variations in analytical procedures [3, 36]. Moreover, processing methods, such as the temperature and drying time of DDGS may vary widely between ethanol producing plants. These are known to be the factors contributing to increased neutral detergent-insoluble CP and acid detergent-insoluble CP, and therefore contributes to the variability in solubility of CP in the rumen [21, 37]. However, information on the details of the feeds used, and the processing technologies applied in the plants, was not available for the DDGS co-products.

## Conclusions

The ruminal CP degradation of the DDGS co-products varied considerably in the present study, which could have been caused by variations in both the quality of the DDGS co-products and the production technology of the individual processing plants. This study showed that the observed UIP content (% of CP) was as follows: HP-DDGS (71.4 %) > corn DDGS (57.5 %) > wheat DDGS (28.8 %) > soybean meal (26.1 %), showing that HP-DDGS and corn DDGS are a good source of UIP. This information can be used as basic data for a more accurate formulation of rations for beef and dairy cattle.

## Abbreviations

ADF, acid-detergent fiber; CP, crude protein; DDGS, dried distillers grains plus solubles; DIP, degraded intake protein; DM, dry matter; ED, effective ruminal degradability; EE, ether extract; HP-DDGS, high-protein distillers grains plus solubles; NDF, neutral-detergent fiber; NPN, non-protein nitrogen; SBM, soybean meal; UIP, undegraded intake protein
